# Hip Pain in The Paediatric Age Group - Transient Synovitis Versus Septic Arthritis

**DOI:** 10.5704/MOJ.1307.011

**Published:** 2013-07

**Authors:** GT Tay, M Ashik, B Tow, Kevin BL Lim

**Affiliations:** Department of Orthopaedic Surgery, KK Women's and Children's Hospital, Singapore; Department of Orthopaedic Surgery, KK Women's and Children's Hospital, Singapore; Department of Orthopaedic Surgery, KK Women's and Children's Hospital, Singapore; Department of Orthopaedic Surgery, KK Women's and Children's Hospital, Singapore

## Abstract

Hip pain, in the paediatric population, is a common complaint and a cause for admission. There are several diagnoses one has to keep in mind, when making the differential diagnosis, as the treatment for each can be quite different. Often, the radiographs obtained for these patients turn up normal. Hence one has to rely on clinical findings, laboratory parameters, and possibly an ultrasound investigation in order to clinch the diagnosis and initiate the appropriate treatment.

**Key Words:**

Paediatric, hip, pain, normal radiographs

## Introduction

Hip pain is a common cause of paediatric admission in our hospital. In this study, we aim to study the causes of the acute hip pain that precipitates admission. We will also delineate the key features of transient synovitis so as to differentiate it from the other possible causes of acute hip pain in children, in particular septic arthritis.

## Materials and Methods

We conducted a retrospective review of paediatric patients admitted over 7 years from 1997-2003, for hip pain, regardless of the diagnosis. Inclusion criteria were extended to patients with any of the following: hip, knee, or thigh pain; pain referred from the hip; a limp; decreased range of motion of the hip; or patients who required admission despite having normal radiographs. Excluded from this study were patients with the following: a radiographic abnormality (e.g. Perthes, SCFE, and DDH); a prior admission for a hip pathology; a known hip abnormality; or known to be on outpatient followup.

The variables analysed included: the patients’ demographic data (age, race), the original as well as final diagnosis, the duration of symptoms tolerated before presentation, history of previous trauma, presence of fever (with record of the maximum temperature), history of an upper respiratory tract infection, weight-bearing status at presentation, past medical history (if any), and the duration of hospitalization. The results of investigations ordered such as white blood cell count, ESR, CRP, ultrasound findings and the presence, as well as size, of effusion, blood cultures, ANA, rheumatoid factor, and anti-streptolysin O titres were also analysed.

## Results

The total number of patients recruited into our study over the span of these seven years was 162. Of these, 120 were male and 42 female. There were 102 Chinese, 20 Indians, 31 Malays and 9 belonging to a minority race. The ages ranged from six months to 14 years.

The principal diagnoses on admission were that of transient synovitis, septic arthritis and hip contusion. The final diagnoses were: 110 (67%) transient synovitis, 13 (8%) hip contusion, 11 (5.2%) septic arthritis of the hip, and 28 (17.9%) miscellaneous different causes (Table I).

The 110 patients with transient synovitis had a mean and median age of 5 years. The average duration of symptoms before presentation spanned 2.7 days. The mean duration of hospital stay was 2 days. There was an accompanying fever in 21% and a preceding URTI in 29% of these patients; 52% were able to weight-bear on the affected limb. The highest white cell count recorded was 10.8 x 10^3^, highest ESR was 16 mm/hr, and highest CRP was 10.18. Ultrasound investigation was performed in 57 of the 110 patients. Of these, 47 (82.5%) showed an effusion, with the mean size of the effusion being 6.53mm. All recovered well.

There were 11 patients with septic arthritis. These patients had a mean age of six years, and a median age of 5 years. The mean duration of symptoms before presentation was 2.5 days and fever was present in all of the patients. The mean maximum temperature was 39.12 degrees Celsius. None of these patients had a preceding upper respiratory tract infection and all were not able to bear weight on the affected limb. The highest white cell count recorded in these patients with septic arthritis was 15.13 x 10³; the highest ESR was 105 mm/hr and the highest C-Reactive Problem (CRP) 116.6. The mean duration of stay of these patients with septic arthritis was 15 days. Ultrasound investigation was performed in all the patients. All ultrasound examinations revealed an effusion with a mean size of 7.6mm.

Despite surgical drainage of the septic arthritis, three patients went on to develop complications. One patient developed cloxacillin-induced hepatitis, another ended up with joint destruction evident a year later, and the third had a subluxable hip which was also evident a year later.

## Discussion

Transient synovitis of the hip is one of the commonest causes of hip pain and a limp in young children. The exact cause is unknown 1. Not all patients with transient synovitis have appreciable effusion on ultrasound, and this parameter on its own does not distinguish it from septic arthritis 2. However, an ultrasound examination that is negative for effusion does suggest that the diagnosis is unlikely to be that of septic arthritis. There are no sonographic signs which can be used to differentiate a sterile, purulent or hemorrhagic effusion 3.

In comparing transient synovitis and septic arthritis, the ages of children at presentation were nearly the same. The durations of symptoms of both conditions were almost identical. Fever was present in 100% of patients with septic arthritis as opposed to 21% of patients with transient synovitis. Upper respiratory tract infection did not precede patients with septic arthritis while 29% of patients with transient synovitis had a preceding upper respiratory infection. All patients with septic arthritis were unable to weight-bear compared to 52% of patients with transient synovitis who were still able to weight-bear. The mean maximum temperature of the patient with septic arthritis was 39.12 as opposed to patients with transient synovitis who were mostly afebrile with a mean maximum temperature of 37.2 degrees. The white cell count, ESR and CRP were also markedly raised in patients with septic arthritis compared to those in patients with transient synovitis. All patients with septic arthritis demonstrated effusion on ultrasound examination while only 82.5% of patients with transient synovitis had effusion on ultrasound examination. However this percentage may not totally reflect the presence of effusion in patients with transient synovitis as only 57 out of the total of 110 patients with transient synovitis underwent ultrasound examination. The sizes of the effusion in both conditions were similar (7.6mm vs 6.5mm). Therefore the ultrasound finding of the absence of an effusion is only useful as a negative predictor of septic arthritis. Ultrasound examination is not a useful diagnostic tool in the evaluation of a painful hip in a paediatric patient.

In conclusion, the commonest diagnosis in the paediatric patient with hip pain and a normal radiograph was transient synovitis. This diagnosis made up 67.9% of the patients recruited. The other most important differential diagnosis in these children is septic arthritis, which will have significant complications and sequelae if missed. Transient synovitis is therefore a diagnosis of exclusion. The tetrad of fever, inability to weight bear, raised white cell count, and raised ESR is paramount in making the diagnosis of septic arthritis, as previously demonstrated by Kocher et al. CRP serves as an independent factor that when raised draws our attention to an infectious cause^4^,^5^. The use of an ultrasound and presence or absence of an effusion is useful as a negative indicator, rather than as a diagnostic tool per se^6^.

**Table I:**
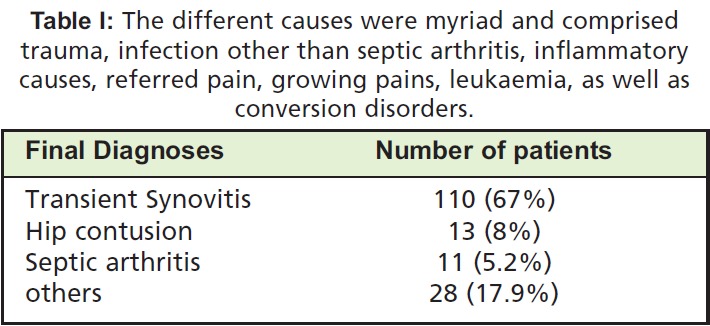
: The different causes were myriad and comprised trauma, infection other than septic arthritis, inflammatory causes, referred pain, growing pains, leukaemia, as well as conversion disorders.
